# Neuralgia of the Glossopharyngeal Nerve in a Patient with Posttonsillectomy Scarring: Recovery after Local Infiltration of Procaine—Case Report and Pathophysiologic Discussion

**DOI:** 10.1155/2015/560546

**Published:** 2015-04-16

**Authors:** L. Fischer, S. M. Ludin, K. Puente de la Vega, M. Sturzenegger

**Affiliations:** ^1^Department of Neural Therapy, IKOM, University of Bern, 3010 Bern, Switzerland; ^2^Department of Neurology, University Hospital of Bern, 3010 Bern, Switzerland

## Abstract

We describe a patient with a three-year history of severe progressive left-sided glossopharyngeal neuralgia (GPN) that failed to adequately respond to various drug therapies. The application of lidocaine spray to the posterior pharyngeal wall provided no more than short-term relief. Apart from a large hypertrophic tonsillectomy scar on the left side all clinical and radiologic findings were normal. In terms of therapeutic local anaesthesia, the hypertrophic tonsillectomy scar tissue was completely infiltrated with the local anaesthetic (LA) procaine 1%. The patient has been almost completely pain-free ever since, and the lidocaine spray is no longer needed. Six weeks after the first treatment a repeat infiltration of the tonsillectomy scar led to the complete resolution of all symptoms. The patient has become totally symptom-free without the need to take any medication now for two and a half years. This is the first report of a successful therapeutic infiltration of a tonsillectomy scar using an LA in a patient with GPN that has been refractory to medical treatment for several years. A possible explanation may be that the positive feedback loop maintaining neurogenic inflammation is disrupted and “sympathetically maintained pain” resolved by LA infiltration.

## 1. Introduction

Glossopharyngeal neuralgia (GPN) is a facial pain syndrome that is characterised by paroxysms of excruciating pain in the sensory area innervated by the auricular and pharyngeal branches of the glossopharyngeal nerve (ninth cranial nerve) and the vagus nerve (tenth cranial nerve) [1]. Typically, affected patients describe a unilateral shooting or lancinating pain felt in the back of the throat in the tonsillar area that can sometimes radiate to the ear. GPN is often missed or misdiagnosed as trigeminal neuralgia (TN) [1].

*Epidemiology*. GPN is a rare condition; the estimated incidence is reported to be in the range of 0.2 to 0.7 per 100,000 patients [2]. GPN is responsible for approximately 0.2 to 1.3% of all cases of facial pain syndromes [2] and has a peak incidence in the fifth and sixth decades of life. GPN, other than TN, affects both women and men equally [2, 3]. In most patients, the pain occurs unilaterally with the left side being more commonly affected than the right [1, 3], but bilateral symptoms are observed in about 25% of the patients and are thus much more common than in patients suffering from TN [2].

*Symptomatology*. The clinical picture is characterised by attacks of flash-like, shooting, stabbing, excruciating pain that patients describe as electric shock- or knife- or pinprick-like sensations. Some patients can experience at the same time dull persistent pain that may also feel like pressure or burning.

Usually, the pain is localised in the back part of the tongue, the tonsils, the pharynx, the larynx, the middle ear, and the angle of the mandible (gonial angle) [1].

Shooting pain can occur spontaneously but is usually triggered by specific stimulating actions such as swallowing, chewing, talking, laughing, yawning, sneezing, brushing teeth or blowing the nose, or touching the ear and the back of the neck. A cough may occur as both a trigger and an accompanying symptom [3]. In some patients, the pain can also be triggered by sweet, sour, cold, or hot food [1].

Normally the pain attacks last from a few seconds to no more than two minutes, the length of the intervals between the attacks varying from several minutes to hours. The frequency of attacks is variable, but the number of attacks can amount to as many as several hundred a day. Pain attacks usually take place during the daytime; however, they may also awaken patients at night.

During the pain attacks, about 10% of the patients also experience severe vagal symptoms that may lead to bradycardia, hypotension, syncope, seizures, or even cardiac arrest [1, 3, 4], which can be explained by the specific fibre composition in the glossopharyngeal nerve (see below).

*Aetiology and Pathogenesis*. The vast majority of GPN are idiopathic; that is, they have no definable cause, and the findings of the clinical examination of the head and the neck are normal. In most cases, imaging studies (including CT and MRI) will also yield results within normal limits.

Rarely, GPN can occur secondary to a definable cause; the glossopharyngeal nerve may, for example, be compressed by adjacent vascular structures, a tumour, an abscess, or abnormal bony structures in the posterior cranial fossa or in the nasopharyngeal space (parapharyngeal tumours or abscesses, a calcified stylohyoid ligament, an elongated long styloid process (so-called Eagle's syndrome), in the context of Paget's disease) [1, 5]. Damage caused by inflammation, for example, in patients with multiple sclerosis or Sjögren's syndrome, has also been reported [1, 5]. Neuromas arising from the ninth cranial nerve are rare and generally almost asymptomatic [6].

*Anatomy of the Glossopharyngeal Nerve*. Developmentally, the glossopharyngeal nerve (ninth cranial nerve) is the nerve of the third pharyngeal (branchial) arch. It exits the skull through the jugular foramen and courses behind the stylopharyngeal muscle, moving distally. It receives sensory fibres from the posterior one-third of the tongue, the pharyngeal mucosa, the tonsils, and the middle ear, and it supplies motor fibres to, among others, the stylopharyngeal muscle, which is responsible for elevating the soft palate during speech and swallowing and is involved in producing the pharyngeal reflex. In addition to sympathetic components, the autonomic fibres of the glossopharyngeal nerve also comprise afferent fibres from the carotid sinus baroreceptors and the chemoreceptors in the carotid body (regulating blood pressure and breathing) as well as parasympathetic secretomotor fibres supplying the parotid gland.

*Diagnosis and Differential Diagnosis*. The diagnosis of GPN is made based on thorough history-taking and a careful clinical examination. The diagnostic criteria for classical GPN were defined in 2013 by the International Headache Society and have been summarised as follows (also see [7]).

*Glossopharyngeal Neuralgia*,* Diagnostic Criteria* (acc. to Headache Classification Committee of the International Headache Society (IHS), 2013 [7]).

- at least three attacks of unilateral pain fulfilling criteria (B) and (C);
- pain is located in the posterior part of the tongue, tonsillar fossa, or pharynx, beneath the angle of the lower jaw, and/or in the ear;
- pain has at least three of the following four characteristics:
  1. recurring in paroxysmal attacks lasting from a few seconds to 2 minutes;
  2. severe intensity;
  3. shooting, stabbing, or sharp in quality;
  4. precipitated by swallowing, coughing, talking, or yawning;
- no clinically evident neurological deficit;
- not better accounted for by another ICHD-3 diagnosis.

Anaesthesia of the posterior pharyngeal wall, for example, using lidocaine spray, followed by the temporary resolution of symptoms is diagnostic of GPN [1, 8]. Imaging studies and, as appropriate, liquor analysis are used to exclude or confirm secondary GPN.

From the point of view of differential diagnosis, GPN must, in particular, be distinguished from trigeminal neuralgia, intermediate nerve neuralgia, and superior laryngeal neuralgia.

*Treatment Options*. In analogy to the treatment of the much more common trigeminal neuralgia, a therapeutic trial with anticonvulsant drugs (carbamazepine, gabapentin, oxcarbazepine, lamotrigine, or phenytoin) is recommended in the first place; however, the evidence base for such an approach is very poor, and, in particular, the conservative long-term therapy has often proved to be difficult and unsatisfactory [3, 5]. The usual painkillers are ineffective, whereas antidepressants such as, for example, amitriptyline, alone or in combination with an antiepileptic drug, can be useful [1].

Invasive treatments, microvascular decompression, intracranial partial rhizotomy, CT-guided radiofrequency thermocoagulation, stereotactic tractotomy/nucleotomy of the pars caudalis of the nucleus tractus spinalis nervi trigemini with radiofrequency lesioning, and Gamma knife surgery, can achieve successful results, especially in the immediate postoperative period, but in many cases symptoms seem to be recurring over time [9–11]. Furthermore, serious complications may occur with invasive procedures [3]. Microvascular decompression seems to be associated with a higher surgical risk in GPN than in TN patients [3].

In cases of inadequate response to medical treatment the additional block of the glossopharyngeal nerve can lead to substantial improvement, as different authors have described in the past few years. Nerve blocking can be achieved using nonneurolytic substances (LA alone and with adjuncts such as steroids, ketamine, etc. [12–14]) or neurolytic substances (phenol, alcohol, and glycerol [15]). The nerve is reached by an intra- or extraoral access.

Various case reports from recent years have shown that in refractory GPN treatment success can also be brought about by “pulsed radiofrequency neurolysis” [16].

## 2. Case Report

### 2.1. History and Findings

During his initial visit the 56-year-old male patient reported that for three years he has been increasingly suffering from a persistent dull, aching pain in the left tonsillar region, which is nonradiating. The patient is unaware of any precipitating event. In addition to this progressive, persistent pain he is afflicted with episodes of sharp, shooting pain that usually lasts from one to 15 minutes and that is triggered by eating, drinking, talking, yawning, and brushing teeth. Preauricular pressure also exacerbates symptoms, which is why he can no longer sleep on his left side. In addition, the pain is worsened by negative emotions and stress.

Long-term treatment with lamotrigine, 2 × 50 mg, and oxcarbazepine, 2 × 375 mg, provided inadequate pain relief, and a pulse steroid therapy, which the patient received one and a half years ago, had no effect whatsoever.

In an e-mail to his neurologist the desperate patient summarised his situation as follows: “Now I muddle through the day, with lidocaine spray, which I apply twice or thrice hourly (every five minutes during meals, and about every two hours at night). Within two minutes, the pain will then disappear almost completely. This thing really takes it out of me (mainly soft, and often tasteless, foods; weight loss; bad moods, etc.).”

Otherwise, the patient is healthy, both physically and emotionally, and, other than the above mentioned drugs, he does not use any medication.

Apart from a tonsillectomy at the age of 9, the repair of a left inguinal hernia, and a varicocele surgery some years ago his medical history-taking revealed no other abnormalities.

The patient had previously been assessed by two neurologists and an ear, nose, and throat specialist; their examinations showed no specific findings. The dental examination (including an orthopantomography and an assessment of the temporomandibular joints) did not show any abnormality either. High-field (3 Tesla) MRI imaging of the neurocranium yielded normal findings that did not detect a morphological substrate and especially gave no indication of a mass lesion, a vessel looping around the nerve, inflammatory changes in the brain stem, vascular variants, or an aneurysm. Neither of the two gonial angles showed signs of irritation.

The clinical examination revealed a large hypertrophic posttonsillectomy scar (left more than right) that seemed to exert traction on the mucosa.

### 2.2. Diagnosis

Idiopathic glossopharyngeal neuralgia on the left side.

### 2.3. Treatment and Future Course

On his initial visit, the patient's hypertrophic tonsillectomy scar was directly infiltrated with 1.5 mL of the LA procaine 1% (without additives); the infiltration was performed using a syringe (Uniject K) with a very fine needle (0.3 × 23 mm).

In less than a minute after infiltration the patient felt completely pain-free, and quite unlike the application of lidocaine spray the procaine infiltration achieved permanent freedom from pain (with respect to both the patient's persistent pain and the episodes of shooting pain).

Six weeks later, when the patient returned for his follow-up visit, he reported that he was still virtually pain-free and that he had reduced his medication dosage by 50%. Since the infiltration of his tonsillectomy scar he had never used the lidocaine spray again.

Subsequently, the infiltration was repeated as described above; the patient continued to be pain-free, except for a week when he experienced low-level pain which did not require the application of lidocaine spray, though.

After another six weeks the patient reported a “perfect outcome.” Eating, teeth cleaning, talking, and so forth caused him no pain any more. He had stopped taking any pain medication, and treatment was no longer needed. The patient has continued to be pain-free until the present day (two and a half years).

### 2.4. Adverse Effects

No adverse effects were observed, except a mild dizziness that appeared right after the injection and lasted a few minutes.

## 3. Discussion

Based on the patient's medical history we assumed that his neuropathic pain was triggered by posttonsillectomy scar tissue in response to irritation from traction (e.g., when talking or swallowing) or pressure/touch (e.g., by food or by lying on the affected side). Scars contain large amounts of collagen. Collagen fibres behave similar to electrical dipoles. In 1974, Athenstaedt demonstrated that electrical signals can be registered upon any mechanical change in length or any deformation of collagen fibres [17]. We think it may be possible that action potentials are transmitted centrally via nociceptive afferent fibres of the ninth cranial nerve when the traction or the pressure exerted on collagen fibres in scar tissue changes. Another possible source for action potentials (ectopic generation) is neuromas in the scar tissue. These mechanisms can lead, as in any chronic or repetitive nociceptive stimulus, to peripheral (e.g., release of substance P) and central sensitisation processes. Here, the autonomic nervous system and, in particular, its sympathetic branch, have a major role in maintaining positive feedback loops [18, 19], which enhance these sensitisation processes [19].

An important factor in the development of these iterative loops is the so-called sympathetic-afferent coupling [20–24]. In certain pathological conditions, sensory coupling may occur between peripheral sympathetic efferent nerves and afferent nociceptive neurons by producing a kind of short circuit. Nociceptive afferents express adrenergic receptors and can thus become susceptible to norepinephrine [20]; as a result the efferent sympathetic system gets connected with the afferent nociceptive system as well: now, enhanced sympathetic activity causes excitation of nociceptive afferents thus generating the perception of pain. In terms of positive feedback (iteration), additional minor (peripheral or central) stimuli may be sufficient in this situation to evoke severe pain [18]. This mechanism also offers an explanation of the increase in pain that our patient experienced for example, as a result of negative emotions or stress.

Even nonirritated scars, which have been normal electrophysiologically and asymptomatic for decades, can be “activated” by sympathetic-efferent nerve fibres and thus represent a permanent potential source of irritation [25].

Injections of LA have (although partially hypothetical) a favourable effect on positive feedback mechanisms by reducing the ectopic generation of action potentials [26] and the peripheral sensitisation (e.g., release of substance P) [27], by reducing or eliminating engramming in the sympathetic nerve system [28], by reducing the activity of the sympathetically activated wide dynamic range (WDR) neurons [29] and by disrupting neuronal reflex arcs. Subsequently they lead to a new organisation (self-organisation) of the pain-processing systems [18, 19].

Even in patients with chronic pain it is possible that the effects of plastic changes with a central state of hyperexcitability, which have been caused by irritation of or damage to peripheral nerves, can be reversed by blocking the spontaneous activity of sensitised nociceptive afferent neurons with an LA [30]. Such a “desensitisation” or even “extinction” of modulating or pain-enhancing signals is a critical step in the management of pain [31].

The fact that procaine infiltration, as we have observed, is helpful in generally softening even hard and hypertrophic scars and thus subsequently attenuates the effects of the traction and pressure mechanisms of collagen [17] may also have contributed to reducing the irritation caused by the scar.

Due to the pathomechanisms that include the sympathetic nerve system, another option might be considered, namely, the therapeutic injection into the superior cervical ganglion. This approach, however, was not required in the case of the patient described because the peripheral “reset” at the location of pain generation had led to complete freedom from pain.

Finally, procaine has been reported to exhibit membrane-stabilising, antiarrhythmic, muscle-relaxing, spasmolytic, perfusion-enhancing, antihistaminic, anti-inflammatory, sympatholytic, parasympatholytic, vasodilator, and DNA-demethylating effects [27, 32, 33]. It contains a carboxylic ester group, its duration of action is no more than approximately 20 minutes and it is known for its limited diffusibility. This is why procaine precisely exerts its effect at the site of injection.

GPN can have a cyclical course with pain-free intervals. This did not apply, though, in the case of our patient, who had been suffering from progressive, chronic pain for three years; also the superimposed stabbing pain was continuously present. The current and lasting freedom from pain, which our patient has experienced after procaine had been injected into his tonsillectomy scar, can thus unambiguously be attributed to the intervention and not to the fact that his GPN followed a cyclical course.

We assume that this is the first description of a successful treatment of refractory GPN by infiltration of a tonsillectomy scar with an LA. Mascolo et al. were the only ones to publish a similar case, however not a patient with infiltration of a scar but of the soft palate [34]. They describe a male patient with GPN that failed to respond to medical drug treatment for several months. Prior to considering surgical therapy, infiltration of bupivacaine 0.25% into the left side of the soft palate and into the trigger points at the base of the palatal arch was performed as a final attempt. Immediately after the injection, the patient was totally pain-free, just like our patient. Therapeutic local anaesthesia was repeated when the pain reappeared 12 days after infiltration. After the second treatment the pain disappeared completely. The authors suggested that the most likely mechanism underlying this treatment success was a disruption of the “sympathetic-sensory coupling.”

## 4. Conclusion

If the standard diagnostic test for GPN, that is, the application of lidocaine spray, is positive, we recommend the following.

1. In tonsillectomised patients, inject an LA (procaine as the drug of first choice) directly into the scar.
2. In patients who still have their tonsils thoroughly infiltrate the soft palate and the posterior pharyngeal wall (in a depth of approximately 2 mm, using a fine needle), before surgical and/or interventional procedures with uncertain therapeutic value and potential complications are planned.

These injections are simple and, if performed* lege artis*, a low-risk management of pain. Studies are required to explore whether these findings can be generalised beyond the setting described.
